# Assessment of the Clinical Usefulness of Preoperative Computed Tomography in Colorectal Cancer Patients Who Received Unplanned Reoperation

**DOI:** 10.1155/2020/6062414

**Published:** 2020-06-27

**Authors:** Rui Wang, Yi Gao, Jia-Yi Li, Zhong-Hui Wang, Qin-qing Li, Jun Feng, Chengde Liao

**Affiliations:** ^1^Radiology Department, Yunnan Cancer Hospital/Third Affiliated Hospital of Kunming Medical University, China; ^2^Colorectal Surgery Department, Yunnan Cancer Hospital/Third Affiliated Hospital of Kunming Medical University, China; ^3^Anesthesiology Department, Yunnan Cancer Hospital/Third Affiliated Hospital of Kunming Medical University, China

## Abstract

**Background:**

In the unplanned reoperation of colorectal cancer patients, computed tomography (CT) is increasingly utilized to locate postoperative complications and previously unlocalized lesions. The purpose of this study is to explore the application of CT in the mortality and complications of the reoperation of colorectal cancer. *Patients and Methods.* We performed a retrospective review of collected data from the colorectal surgeries of 90 identified colorectal cancer patients who received an unplanned reoperation from 2010 to 2018. Patients were stratified according to those with preoperative CT imaging (CT group, *n* = 36) and those without preoperative CT imaging (NCT group, *n* = 54). Twenty-four statistical indicators of each patient were studied, including their preoperative risk, surgical characteristics, and postoperative outcomes, and satisfaction was evaluated. All data were statistically analysed for predicting postoperative complications by univariate and multivariate logistic regression analyses.

**Results:**

Ninety patients received an unplanned reoperation in the study, and 40% (36/90) of these patients underwent preoperative CT examination. Patients' risk factors were similar between CT and NCT groups. Preoperative imaging was more commonly performed for reoperative new anastomosis + ileostomy but less common for reoperative Dixon's procedure. The operative duration of the NCT group was longer (139 vs. 104 min, respectively, *P* = 0.01). Preoperative NCT examination (OR 1.24; 95% CI = 1.09‐1.42; *P* = 0.01) was an independent predictor of postoperative complications. Importantly, three patients died after an unplanned reoperation for colorectal cancer, which occurred only in the NCT group (5.6% vs. 0.0%, *P* = 0.01).

**Conclusion:**

The use of conventional preoperative CT optimizes the choice of the surgical site and the strategy of laparotomy, so as to reduce the length of operation. Preoperative imaging evaluation should be performed for patients undergoing repeat abdominal surgery.

## 1. Introduction

Colorectal cancer (CRC) is one of the most common cancers worldwide. According to the data of the China Cancer Registration Center, the incidence of colorectal cancer in China was 26.90/100,000 in 2013, and the mortality rate was 13.03/100,000 [1]. The outcomes of CRC vary widely, depending on both the patients' and tumors' characteristics, as well as the quality of treatments administered [2]. Unfortunately, various complications often occur after an operation, which may lead to the need for a reoperation. An unplanned reoperation within 30 days after colorectal cancer surgery not only leads to the prolongation of hospitalization time but also increases the operative risk of mortality. Thus, authors and policymakers have use unplanned reoperation rates as a quality metric for monitoring quality across hospitals [3].

Unplanned reoperation was defined by the US NSQIP database as an “unplanned return to the operating room for a surgical procedure related to either the index or concurrent procedure performed” [4]. Earlier studies have found that colorectal operations account for the greatest proportion of unplanned reoperations. From 2003 to 2013, 6.7% of the patients who had colorectal cancer surgery underwent a reoperation in the NSQIP database (178 cases) [5]. The main purpose of a reoperation is to resolve the complications caused by (or related to) the first operation, including hemostasis, fistula closure, and adequate drainage [6].

The safety of an unplanned reoperation depends largely on the preoperative evaluation. Compared with physical examination and blood tests, medical imaging is of great significance to the safety and outcome of the surgery [7]. At present, commonly used imaging modalities include ultrasound (US), computed tomography (CT), and endoscopic ultrasonography. The imaging value chain is even being used in most of the patients when they are diagnosed, and its use in an unplanned reoperation has been underestimated in the past decades. For the colorectal cancer patients who had to undergo an unplanned reoperation, CT allows doctors to see the anatomical structure of the colon and rectum, to better define the types of complications, and to aid surgical decision-making [8, 9]. Despite the clear implications of an unplanned reoperation on patients' outcomes and costs, there are no studies focused on the benefits of imaging examination for these colorectal cancer patients.

The objectives of this study were to characterize the use of preoperative CT imaging for reoperation in patients with colorectal cancer and to examine its role in operative mortality and complications.

## 2. Methods

### 2.1. Patients

The Yunnan Tumor Hospital's Institutional Review Board (IRB) waived the requirement for written informed consent. Using the database of colorectal surgery in the Third Affiliated Hospital of Kunming Medical University, a retrospective study was conducted on all patients who had undergone abdominal surgery (January 2010 to December 2018). These patients had undergone colorectal tumor resection before. Individual radiologic reports were reviewed to determine preoperative CT findings. Patients were divided into two groups: the preoperative routine CT (CT) group and the preoperative non-routine CT (NCT) group. Patients undergoing small bowel transplantation, removal or placement of an enterostomy device, or laparoscopic surgery were excluded from the analysis. The established colorectal surgery database definition is used for all preoperative variables, postoperative complications, and prognosis. All interesting patient outcomes are predetermined before data collection. Operative mortality was defined as patient deaths occurring prior to hospital discharge or within 30 days of operation. Major complications included the composite incidence of postoperative mortality, anastomotic leakage, intestinal obstruction, and abdominal abscess.

### 2.2. Statistical Analysis

Statistical analyses of all data were performed using SPSS software, version 23 (SPSS, Inc., Chicago, IL, USA). The primary results were the incidence of operative mortality and postoperative complications. The appropriate hypothesis test was used to determine the characteristics of patients and surgeries between the study groups and the observation differences of results. According to the comparison between the operation results of the patients and the previous CT results, if they are consistent, they are true positive, if not, they are false positive. Categorical variables were compared using either Pearson's chi-squared test or Fisher's exact tests, and continuous variables were compared by Student's *t*-test for normally distributed data or Mann-Whitney's *U*-test for non-normally distributed data where appropriate [10]. All categorical variables were expressed as percentage within the group, and continuous variables were expressed as mean ± standard deviation (S.D.) or median (interquartile range). All the *P* values reported were two-tailed, and statistical significance was indicated by *P* < 0.05.

## 3. Results

### 3.1. Comparison of Patient Characteristics and Preoperative Risk Factors

Univariate analysis of risk factors for all colorectal cancer patients is shown in Table 1. Overall, there were 5845 cases of colorectal cancer undergoing major abdominal surgery between 2010 and 2018. Among these, 5755 cases were excluded from the present analysis because of no unplanned reoperation. Patients who were excluded did not significantly differ from patients included in the analysis by age, sex, BMI index, and disease staging. However, there were differences in some preoperative clinical variables between the excluded patients and the analyzed patients, such as distant cancer and primary resection type. Although the differences were statistically significant, there was no clinical significance associated with the exclusion of these patients.

Of the 90 patients included within this cohort, 36 patients underwent colorectal cancer reoperation with preoperative CT imaging, and 54 patients underwent surgery without preoperative CT imaging (Table 2). The average age of patients in the CT group and the NCT group was similar. Compared with the NCT group (33.3%), there were slightly more female patients in the CT group (38.9%), but the difference was not statistically significant. Among the main risk factors of colorectal surgery in the CT group, the factors for the primary resection type include right sided (25.0%), left sided (19.4%), rectal (50.0%), and total colectomy (5.6%). The choice of the primary resection type was similar between the CT and NCT groups.

### 3.2. Comparison of Operative Features for CT and NCT Patients

Operative features for patients undergoing colorectal cancer reoperations are detailed in Table 3. The performance of new anastomosis + ileostomy operations occurred in 33.3% of CT patients and in 18.5% of NCT patients (*P* = 0.01). The proportion of NCT patients receiving Dixon's procedure was higher (9.3% vs. 2.8%, *P* = 0.05). Other surgical procedures were similar between the study groups. CT patients underwent a higher percentage of emergency operations (86.1% vs. 74.1%, *P* = 0.03). Performance of elective surgery occurred in 13.9% of CT patients and in 25.9% of NCT patients. As anticipated, patients in the NCT group, compared with those in the CT group, had a greater duration of operation (135 min vs. 101 min, respectively, *P* = 0.01). Despite the preoperative use of CT imaging, there was no significant difference between the study groups in the amount of intraoperative blood transfusion and bleeding.

### 3.3. Comparison of Postoperative Outcomes for CT and NCT Patients

There was little difference in the postoperative results observed between the study groups (Table 4). The incidence of postoperative anastomotic leakage was the highest in the CT group and the NCT group among all complications (16.7% vs. 18.5%, *P* = 0.74). In addition, other complications occurred between the study groups; however, this trend did not reach statistical significance. Similarly, incrementally higher rates of postoperative anastomotic leakage, abdominal abscess, rupture after laparotomy, cardiac symptoms, and pancreatic fistula were observed for NCT patients. However, postoperative death occurred only in the NCT group (5.6% vs. 0.0%, *P* = 0.01).

### 3.4. Radiologic Findings of Patients with Unplanned Reoperation for Colorectal Cancer

The imaging findings associated with 36 reoperative events in patients with colorectal cancer are shown in Table 5. The most common imaging manifestation was anastomotic leakage in 10 cases (27.8%). An unusual radiologic finding was intestinal adhesion seen in patients prior to 9 additional surgical interventions (25.0%). Ascites occurred in 8 patients (22.2%). Abnormalities associated with the abdominal wall or the undersurface of the hemidiaphragms was seen in 6 cases (16.7%). No radiologic findings were found in 7 surgical interventions (19.4%). In 5 patients, there was no radiologic abnormality due to the second examination and occasional indications. Therefore, in the absence of positive imaging results, only 2 interventions were performed to patients with symptoms.

### 3.5. Predictors of Postoperative Complication Involvement

The results of univariable and multivariable analyses for postoperative complication involvement are shown in Table 6. In the table, “Ref” (reference) means the reference value compared with other indicators (OR = 1). In univariate analysis, patients with postoperative complications may have a later stage of disease (*P* < 0.05). In addition, the risk factors of postoperative complications are left colectomy (*P* = 0.05), emergency operation status (*P* = 0.03), and preoperative NCT examination (*P* = 0.01). In multivariate analysis, only preoperative NCT examination (OR 1.24; 95%CI = 1.09‐1.42; *P* = 0.01) was an independent predictor of postoperative complications. It should be noted that postoperative complications were not associated with the length of time for reoperation duration (*P* = 0.12).

## 4. Discussion

Reoperative abdominal and pelvic surgery has become increasingly more common in recent years and is associated with elevated patients' morbidity and mortality. The short-term reoperation not only prolongs patients' hospital length of stay and increases their economic burden but also affects the follow-up treatment after colorectal cancer surgery [11]. Abdominal and pelvic reoperation also has a long-term treatment effect and, more importantly, increases the possibility of perioperative mortality. The implications for reoperation are sizable. Earlier data have concluded that compared with non-reoperated patients, those who underwent reoperation within 30 days had worse long-term oncologic outcomes [12]. The reason may be that these cases often present inherent technical challenges of intestinal adhesions and an increased risk of injury upon reentry or to a previously placed double-stapling device [13].

Of note, there is no specific study on imaging evaluation of an unplanned reoperation for rectal cancer patients. The possible complications after the first operation (always the reasons for unplanned operation) include anastomotic leakage, bleeding, intestinal obstruction, and infection [14]. An unplanned reoperation is technically more difficult and has a higher risk. In most clinical reports, surgeons usually decide whether a reoperation is needed based on their own experience, clinical manifestations, drainage, tumor markers, and so on. Most of the patients in our study underwent contrast-enhanced CT examination before reoperation, and the imaging results were highly consistent with clinical features and operative outcomes. Our results supported the benefit of the preoperative imaging examination in these cases. Identification of the reasons for an unplanned reoperation could help stratify more aggressive surgical treatment, including more extensive nodal dissection and even preventive intraperitoneal hyperthermia [15]. In this study, we also defined the efficacy of conventional CT on these patients and identified some risk factors associated with accidental reoperation. Although the colorectal cancer surgeries are quite safe, a range of postoperative complications may occur and postoperative death is an infrequent but real risk. Supported by our results and previous studies, we highlighted the benefits of preoperative CT for the optimal surgical performance.

So far, the diagnostic challenge of an unplanned reoperation for patients with colorectal cancer remains an ongoing problem for the clinician. Meanwhile, the surgeon is caught between the dangers of missing or delaying the diagnosis of reoperation and the risks of nontherapeutic surgery. If CT imaging can truly improve its diagnostic accuracy, it would be a solution to this clinical dilemma. Difficulty in operation easily leads to a longer operative time, and many studies have even proved that operation time is an independent risk factor for anastomotic leakage [16]. Therefore, it is particularly important to correctly evaluate the difficulty of operation previously. Our study shows that the unplanned reoperation duration in the patients who have preoperative CT evaluation is significantly shorter, and even after correcting the clinical data, this difference still exists. It is suggested that a preoperative imaging assessment would greatly shorten the time required for intraoperative exploration. On the other hand, although it cannot reduce the difficulty of surgery, preoperative CT enables surgeons to improve the assessment and reduce the risk of surgery to a reasonable level [17]. Beyond that, CT examination has a high spatial resolution and can accurately display the type and location of complications caused by a previous operation [18]. Some measurement data including bleeding volume, fistula size, and degree of intestinal obstruction are obtained by CT measurement, so that surgeons can predict surgery duration more accurately. The success rate of an operation and the accuracy rate of clinical diagnosis are also greatly improved.

Intestinal fistula is the most common complication after colorectal cancer surgery in this study. The abdominal and pelvic CT scan is a sensitive and noninvasive imaging examination for the suspected patients, which has a diagnostic accuracy of 60% to 100% [19]. Compared with other methods, the advantage of a CT scan lies in its ability to demonstrate accompanying soft tissue masses forming the fistula between the anal tube and the bowel. It can also identify adhesions between the intestine and other abdominal organs, showing abscess formation [20]. Detection of a mass outside the bowel wall, local thickening of the bowel wall, and thickening of the adjacent bowel wall can aid in the localization of fistulae. In particular, observation of a contrast agent in fistulae on the axial or (reconstructed) coronal CT images can help in diagnosing this disease. The most usual CT findings are gas in intestine, local thickening of intestinal wall, thickening of adjacent bowel wall, and adherence of soft tissue masses. As for magnetic resonance imaging (MRI), it is not clear whether MRI offers any significant benefits over a CT scan. Some studies suggest MRI as a second-line investigation modality for the diagnosis of a colovesical fistula and complex fistulae [21].

The incidence of intestinal obstruction is second only to intestinal fistula in this study. Intestinal obstruction after colorectal cancer surgery is a rare condition due to severe gastrointestinal motility disorder. Patients with intestinal obstruction experience symptoms of mechanical obstruction, but reliable clinical signs that may help distinguish between actual mechanical obstruction and intestinal pseudoobstruction are lacking. Additionally, abdominal plain films that commonly show bowel dilatation with air-fluid levels do not reach acceptable degrees of specificity to exclude actual obstruction [22]. Therefore, most patients usually undergo multiple and often fruitless surgery, often leading to repeated bowel resections before diagnosis is made. In these patients who present with abdominal signs mimicking symptoms that would warrant surgical exploration, CT scan is helpful to resolve this diagnostic dilemma. CT shows a diffusely distended bowel and helps to rule out a mechanical cause of obstruction, thus obviating the need for unnecessary laparotomy [23]. In patients with intestinal obstruction after colorectal cancer surgery, CT shows pneumatosis intestinalis, pneumoperitoneum, or intussusception [24]. In addition, for patients with postoperative bleeding, CT examination can also detect the bleeding site of patients with limitations, and accurately calculate the amount of abdominal bleeding, combined with dynamic detection of intestinal bleeding changes in patients, to provide effective reference for diagnosis and treatment. In the treatment of complications after a reoperation for colorectal cancer, laparoscopic surgery was excluded in this study. Although some places will choose laparoscopic surgery as the way of reoperation for colorectal cancer, the proportion of conversion to laparotomy is very high, so it is generally not the first choice [25].

There is no doubt that preoperative CT examination can reduce the risk of surgery, but we still find that some patients did not undergo CT examination in time because of their lack of symptoms or their critical condition, which may greatly affect the surgeon's surgery risk assessment. In our study, not all CT imaging reports are consistent with the intraoperative findings, which may be due to the changes in intestinal anatomy, intestinal distention, pneumoperitoneum, and other reasons; these changes may also affect the surgeons observing the lesions. The existence of a drainage tube and a circular stapler can also lead to inadequate preparation for image examination and the failure of CT examination [26]. In addition, the coexistence of the lesions with these complications makes the correct diagnosis more difficult. Therefore, a junior radiologist should accumulate experience to distinguish normal and abnormal image changes after an operation.

Despite our results, this study has noteworthy limitations. First, the retrospective design introduces inherent selection bias. Second, the admittedly small sample size limits our ability to detect small, statistical differences between CT and non-CT groups. In addition, the heterogeneity of the technique used for the acquisition of CT images performed in different institutions with the use of variable equipment and different technical details and preparations may have accounted for a significant source of bias in our study. Conversely, these results may reflect real-world outcomes and increase the applicability of our findings to current clinical practice. Additionally, CT images were reviewed by two separate radiologists and this may lead to a deviation between observers. Finally, the changes of surgical procedures and imaging methods in the recent ten years have some effects on the results of this study, which cannot be completely ruled out. Nevertheless, the markedly reduced operation duration for patients with preoperative CT imaging provide an important clinical contribution to current colorectal surgical literature.

## 5. Conclusions

In conclusion, an unplanned reoperation is the result of complications after colorectal cancer surgery, and most patients have abnormal imaging changes. Using routine preoperative CT optimizes the choice of the surgical site and opening strategy to minimize operation time and should be performed for patients undergoing reoperative abdominal and pelvic operations. Finally, CT examination can be a good choice for the preoperative evaluation of patients with colorectal cancer undergoing an unplanned reoperation.

## Figures and Tables

**Table 1:** Preoperative and risk factors associated with unplanned reoperations of colorectal cancer.

Factor	Patients, no. (%)			Kruskal gamma coefficient (95% CI)
		Unplanned reoperation		
	Total (*n* = 5845)	Yes (*n* = 90)	No (*n* = 5755)	
Age (y)				
≤40	537 (9.2)	8 (8.9)	529 (9.2)	
41-60	2145 (36.7)	35 (38.9)	2110 (36.7)	0.02 (−0.02 to 0.17)
61-80	2718 (46.5)	43 (47.8)	2675 (46.5)	
≥81	319 (5.5)	4 (4.4)	315 (5.5)	
Sex				
Male	3688 (63.1)	58 (64.4)	3630 (63.1)	
Female	2157 (36.9)	32 (35.6)	2125 (36.9)	0.05 (−0.05 to 0.16)
BMI, median (IQR)	26.8 (6.2)	26.2 (7.3)	26.8 (6.2)	
Disease staging				
I	1040 (17.8)	18 (20)	1022 (17.8)	
II	2069 (35.4)	30 (33.3)	2039 (35.4)	0.13 (−0.06 to 0.42)
III	1759 (30.1)	26 (28.9)	1733 (30.1)	
IV	977 (16.7)	16 (17.8)	961 (16.7)	
Distant cancer				
Yes	590 (10.1)	16 (17.8)	551 (9.6)	0.31 (0.03 to 0.58)
No	5255 (89.9)	74 (82.2)	5204 (90.4)	
Primary resection type				
Right sided	1660 (28.4)	22 (24.4)	1638 (28.5)	
Left sided	1391 (23.8)	18 (20)	1373 (23.8)	0.23 (0.03 to 0.47)
Rectal	2402 (41.1)	46 (51.1)	2356 (40.9)	
Total colectomy	392 (6.7)	4 (4.4)	388 (6.7)	

**Table 2:** Preoperative and risk factors for patients undergoing reoperative colorectal operations without preoperative imaging compared to those with preoperative imaging (*n* = 90).

Factor	Preoperative CT (*n* = 36)	No preoperative CT (*n* = 54)	*P* value
Age (y)			
≤40	3 (8.3%)	5 (9.3%)	0.80
41-60	15 (41.7%)	20 (37.0%)	0.50
61-80	17 (47.2%)	26 (48.1%)	0.90
≥81	1 (2.8%)	3 (5.6%)	0.32
Sex			
Male	22 (61.1%)	36 (66.7%)	0.41
Female	14 (38.9%)	18 (33.3%)	0.41
BMI, median (IQR)	25.8 (7.4)	26.5 (7.3)	—
Disease staging			
I	8 (22.2%)	10 (18.5%)	0.52
II	13 (36.1%)	17 (31.5%)	0.49
III	10 (27.8%)	16 (29.6%)	0.78
IV	5 (13.9%)	11 (20.4%)	0.22
Distant cancer			
Yes	7 (19.4%)	9 (16.7%)	0.62
No	29 (80.6%)	45 (83.3%)	0.62
Primary resection type			
Right sided	9 (25.0%)	13 (24.1%)	0.88
Left sided	7 (19.4%)	11 (20.4%)	0.86
Rectal	18 (50.0%)	28 (51.9%)	0.79
Total colectomy	2 (5.6%)	2 (3.7%)	0.5

**Table 3:** Operative characteristics for patients undergoing reoperative colorectal operations without preoperative imaging compared to those with preoperative imaging (*n* = 90).

Factor	Preoperative CT (*n* = 36)	No preoperative CT (*n* = 54)	*P* value
Reoperation type			
New anastomosis + ileostomy	12 (33.3%)	10 (18.5%)	0.01
New anastomosis without ileostomy	5 (13.9%)	4 (7.4%)	0.14
Repair of anastomosis + ileostomy	7 (19.4%)	13 (24.1%)	0.42
Repair of anastomosis without ileostomy	5 (13.9%)	12 (22.2%)	0.13
Lavage, drainage, ileostomy	6 (16.7%)	10 (18.5%)	0.74
Dixon's procedure	1 (2.8%)	5 (9.3%)	0.05
Elective operative status	5 (13.9%)	14 (25.9%)	0.03
Emergent operative status	31 (86.1%)	40 (74.1%)	0.03
Reoperation duration (min) (median, range)	101 (53-269)	135 (68-302)	0.01
Intraoperative blood products transfused			
Packed red blood cells (units)	2.1 ± 2.2	2.2 ± 2.3	0.77
Fresh frozen plasma (units)	1.2 ± 2.1	1.2 ± 1.8	0.51
Platelets (units)	0.4 ± 0.8	0.4 ± 1.0	0.58
Cryoprecipitate (units)	1.3 ± 0.8	1.4 ± 0.8	0.63
Intraoperative bleeding (units)	3.4 ± 2.8	3.8 ± 2.7	0.81

**Table 4:** Postoperative complications and outcomes for patients undergoing reoperative colorectal operations without preoperative imaging compared to those with preoperative imaging (*n* = 90).

Factor	Preoperative CT (*n* = 36)	No preoperative CT (*n* = 54)	*P* value
Anastomotic leakage	6 (16.7%)	10 (18.5%)	0.74
Intestinal obstruction	4 (11.1%)	6 (11.1%)	>0.99
Wound infection	3 (8.3%)	4 (7.4%)	0.81
Rupture after laparotomy	3 (8.3%)	5 (9.3%)	0.80
Abdominal abscess	2 (5.6%)	4 (7.4%)	0.61
Postoperative bleeding	3 (8.3%)	3 (5.6%)	0.34
Cardiac	1 (2.8%)	2 (3.7%)	0.72
Pulmonary	1 (2.8%)	1 (1.9%)	0.67
Renal	1 (2.8%)	1 (1.9%)	0.67
Hepatic	2 (5.6%)	1 (1.9%)	0.17
Pancreatic fistula	1 (2.8%)	2 (3.7%)	0.72
Operative mortality	0 (0.0%)	3 (5.6%)	0.01

**Table 5:** Radiologic findings associated with 36 reoperative surgeries in patients with colorectal cancer.

Radiologic findings	Number of cases	Rate
Anastomotic leakage	10	27.8%
Intestinal adhesion	9	25.0%
Ascites	8	22.2%
Tumor mass	6	16.7%
Abdominal wall or diaphragmatic irregularities	6	16.7%
Bowel obstruction	5	13.9%
Perihepatic irregularities	5	13.9%
Perisplenic irregularities	4	11.1%
Extrinsic small bowel compression	4	11.1%
Mesenteric thickening	3	8.3%
Lymph nodes enlargement	2	5.6%
No radiologic findings	7	19.4%

**Table 6:** Univariable and multivariable analyses for postoperative complications.

Variable	Univariable analysis		Multivariable analysis	
	OR (95% CI)	*P* value	OR (95% CI)	*P* value
Age (y)				
≤60	0.72 (0.37–1.38)	0.32		
61-80	Ref			
≥81	0.85 (0.35–2.08)	0.72		
Sex				
Male	1.15 (0.61–2.16)	0.68		
Female	Ref			
BMI, median (IQR)	1.03 (0.99–1.08)	0.15		
Disease staging				
I/II	Ref		Ref	
III	3.40 (1.02–11.28)	0.05	2.62 (0.74–9.27)	0.14
IV	8.46 (2.03–35.20)	0.01	2.14 (0.39–11.79)	0.39
Primary resection type				
Right sided	0.54 (0.28–1.05)	0.07	0.56 (0.27–1.15)	0.11
Left sided	1.10 (0.46–2.61)	0.05	1.02 (0.53–3.23)	0.15
Rectal/Total	Ref		Ref	
Emergent operative status				
Yes	2.31 (1.10–4.83)	0.03	1.98 (0.75–5.26)	0.17
Reoperation duration				
Short	Ref			
Long	1.65 (0.89–3.09)	0.12		
Preoperative CT				
No	1.23 (1.08–1.39)	0.01	1.24 (1.09-1.42)	0.01

## Data Availability

The datasets used and/or analysed during the current study are available from the corresponding author on reasonable request.
